# Ulcère herpétique en carte géographique

**DOI:** 10.11604/pamj.2014.18.65.4400

**Published:** 2014-05-19

**Authors:** Amal Alouan, Ouafa Cherkaoui

**Affiliations:** 1 Université Mohammed V Souissi, Service d'Ophtalmologie A de l'hôpital des spécialités, Centre Hospitalier Universitaire, Rabat, Maroc

**Keywords:** Ulcère de la cornée, herpés, rougeur oculaire, Corneal ulcer, herpes, eye redness

## Image en medicine

Patient âgé de 26 ans sans antécédents particuliers qui a présenté 4 jours avant sa consultation une rougeur oculaire accompagnée de douleur et baisse de l'acuité visuelle au niveau de l'OD avec notion d'automédication par corticoïde locale. L'examen ophtalmologique trouve une acuité visuelle réduite à compte les doigts à 1m, une hyperhémie conjonctivale avec cercle périkératique.la cornée est le siège d'un large ulcère géographique avec test à la fluorescéine positive.par ailleurs l'examen est sans anomalies. L'ulcère corné en carte géographique est très caractéristique de l'Herpes simplex virus, la lésion initiale est une ulcération dendritique au départ qui s’étend après corticothérapie .le traitement repose sur les antiviraux locaux avec un traitement préventif au long cours en cas de récidive.

**Figure 1:**
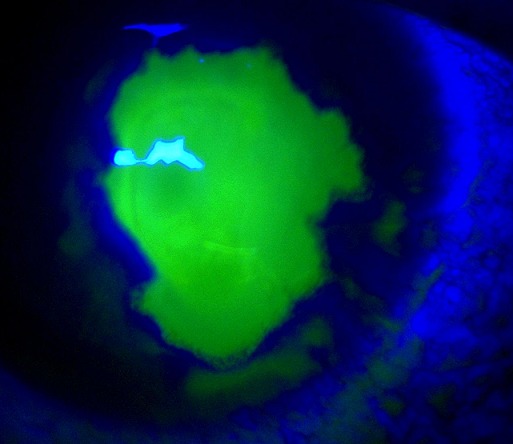
Ulcère de cornée en carte géographique au test à la fluorescéine (coloration verte)

